# Association Between Renin-Angiotensin-Aldosterone System Inhibitors and Clinical Outcomes in Patients With COVID-19: A Systematic Review and Meta-analysis

**DOI:** 10.1001/jamanetworkopen.2021.3594

**Published:** 2021-03-31

**Authors:** Ranu Baral, Vasiliki Tsampasian, Maciej Debski, Brendan Moran, Pankaj Garg, Allan Clark, Vassilios S. Vassiliou

**Affiliations:** 1 Department of Cardiology, Norfolk and Norwich University Hospital, Norwich, United Kingdom; 2 Department of Cardiology, Norwich Medical School, University of East Anglia, Norwich, United Kingdom; 3 National Health Service 111 COVID-19 Clinical Assessment Service, Bicester, United Kingdom; 4 Neasden Medical Centre, London, United Kingdom; 5 Healix International, Esher, United Kingdom; 6 Department of Medical Statistics, Norwich Medical School, University of East Anglia, Norwich, United Kingdom

## Abstract

**Importance:**

The chronic receipt of angiotensin-converting enzyme inhibitors (ACEIs) and angiotensin receptor blockers (ARBs) has been assumed to exacerbate complications associated with COVID-19 and produce worse clinical outcomes.

**Objective:**

To conduct an updated and comprehensive systematic review and meta-analysis comparing mortality and severe adverse events (AEs) associated with receipt vs nonreceipt of ACEIs or ARBs among patients with COVID-19.

**Data Sources:**

PubMed and Embase databases were systematically searched from December 31, 2019, until September 1, 2020.

**Study Selection:**

The meta-analysis included any study design, with the exception of narrative reviews or opinion-based articles, in which COVID-19 was diagnosed through laboratory or radiological test results and in which clinical outcomes (unadjusted or adjusted) associated with COVID-19 were assessed among adult patients (≥18 years) receiving ACEIs or ARBs.

**Data Extraction and Synthesis:**

Three authors independently extracted data on mortality and severe AEs associated with COVID-19. Severe AEs were defined as intensive care unit admission or the need for assisted ventilation. For each outcome, a random-effects model was used to compare the odds ratio (OR) between patients receiving ACEIs or ARBs vs those not receiving ACEIs or ARBs.

**Main Outcomes and Measures:**

Unadjusted and adjusted ORs for mortality and severe AEs associated with COVID-19.

**Results:**

A total of 1788 records from the PubMed and Embase databases were identified; after removal of duplicates, 1664 records were screened, and 71 articles underwent full-text evaluation. Clinical data were pooled from 52 eligible studies (40 cohort studies, 6 case series, 4 case-control studies, 1 randomized clinical trial, and 1 cross-sectional study) enrolling 101 949 total patients, of whom 26 545 (26.0%) were receiving ACEIs or ARBs. When adjusted for covariates, significant reductions in the risk of death (adjusted OR [aOR], 0.57; 95% CI, 0.43-0.76; *P* < .001) and severe AEs (aOR, 0.68; 95% CI, 0.53-0.88; *P* < .001) were found. Unadjusted and adjusted analyses of a subgroup of patients with hypertension indicated decreases in the risk of death (unadjusted OR, 0.66 [95% CI, 0.49-0.91]; *P* = .01; aOR, 0.51 [95% CI, 0.32-0.84]; *P* = .008) and severe AEs (unadjusted OR, 0.70 [95% CI, 0.54-0.91]; *P* = .007; aOR, 0.55 [95% CI, 0.36-0.85]; *P* = .007).

**Conclusions and Relevance:**

In this systematic review and meta-analysis, receipt of ACEIs or ARBs was not associated with a higher risk of multivariable-adjusted mortality and severe AEs among patients with COVID-19 who had either hypertension or multiple comorbidities, supporting the recommendations of medical societies. On the contrary, ACEIs and ARBs may be associated with protective benefits, particularly among patients with hypertension. Future randomized clinical trials are warranted to establish causality.

## Key Points

**Question:**

Is the receipt of angiotensin-converting enzyme inhibitors (ACEIs) or angiotensin receptor blockers (ARBs) associated with worse clinical outcomes among patients with COVID-19?

**Findings:**

In this systematic review and meta-analysis of 52 studies that evaluated clinical outcomes among 101 949 total patients with COVID-19 who did and did not receive ACEIs or ARBs, a significantly lower risk of multivariable-adjusted mortality and severe adverse events was found among patients who received ACEIs or ARBs compared with patients who did not. A subgroup analysis of patients with hypertension indicated significant decreases in mortality and severe adverse events among patients receiving ACEIs or ARBs in both unadjusted and adjusted analyses.

**Meaning:**

The study’s findings suggest that ACEIs and ARBs may be associated with protective benefits for patients with COVID-19 and that patients may continue receiving ACEIs and ARBs for the treatment of any condition without an increased risk of worse outcomes unless specifically advised to avoid them by treating clinicians.

## Teaser

This systematic review and meta-analysis examines the association between the receipt of angiotensin-converting enzyme inhibitors or angiotensin receptor blockers and clinical outcomes among adult patients with coronavirus disease 2019.

## Introduction

Coronavirus disease 2019 (COVID-19), a rapidly evolving pandemic infecting more than 93 million people worldwide to date,^1^ is associated with worse clinical outcomes in patients with existing cardiovascular diseases, including hypertension and diabetes.^2,3^ Renin-angiotensin-aldosterone system (RAAS) inhibitors, specifically angiotensin-converting enzyme inhibitors (ACEIs) and angiotensin receptor blockers (ARBs), which are frequently used for the treatment of cardiovascular conditions, are subjects of debate because angiotensin-converting enzyme 2 acts as a binding site for the virus to gain cellular entry.^4^ This debate has elicited several theories suggesting that the chronic receipt of RAAS inhibitors may exacerbate COVID-19 and produce worse outcomes.^4^

Several observational studies have since evaluated the association of ACEIs and ARBs (ACEIs/ARBs) with clinical outcomes in patients with COVID-19. Although a few studies have reported an increased risk of severe disease,^5,6^ most have found no association^7,8^ or even beneficial associations with the receipt of these drugs.^9,10^ A previous meta-analysis^11^ that examined 16 studies with 28 000 total patients reported no significant association between the receipt of ACEIs/ARBs and mortality or severe adverse events (AEs) among individuals with multiple comorbidities (OR, 0.67; 95% CI, 0.44-1.03; *P* = .07) and a significant association between ACEIs/ARBs and protective benefits among individuals with hypertension (OR, 0.67; 95% CI, 0.50-0.91; *P* = .01). Since the publication of that meta-analysis, more original studies have been published, allowing increased statistical power to further investigate specific subgroups.

Given the increasing number of COVID-19 cases and an evolving second wave of infections, it is important to summarize the data thus far to provide an updated perspective and an understanding of the association between ACEIs/ARBs and clinical COVID-19 outcomes. The inclusion of more studies and patients will allow the identification of more accurate associations with smaller CIs, producing findings that are more likely to represent true associations. In addition, the inclusion of only peer-reviewed studies will reinforce the conclusions. Findings from the present meta-analysis will be relevant for the clinical management of millions of patients receiving these drugs worldwide.^12^

## Methods

### Study Selection

The PubMed and Embase databases were systematically searched from December 31, 2019, until September 1, 2020, for studies published in English. Terms such as *angiotensin-converting enzyme inhibitors*, *angiotensin receptor blockers*, *coronavirus disease 2019*, and *SARS-COV-2* were used for a comprehensive search. Additional details about the search strategy are available in eMethods in the Supplement. The references of retrieved articles were manually screened for relevant studies to expand the search. This study followed the Preferred Reporting Items for Systematic Reviews and Meta-analyses (PRISMA) reporting guideline.

All studies identified in our search were screened by 3 authors (R.B., M.D., and V.T.) using article titles and abstracts. Duplicate studies and multiple reports using the same data were removed. Any article identified as having the potential to fulfill our inclusion criteria underwent full-text evaluation. We included studies meeting the following criteria: (1) any study design, with the exception of narrative reviews and opinion-based articles; (2) adult (≥18 years) study population; (3) participants with COVID-19 diagnosed through laboratory or radiological test results; and (4) assessment of clinical or mortality outcomes (unadjusted or adjusted) among patients receiving ACEIs/ARBs. The mortality and clinical severity data of patients receiving ACEIs/ARBs were compared with those of patients not receiving ACEIs/ARBs.

### Data Extraction and Quality Assessment

Three authors (R.B., V.T., and M.D.) independently extracted relevant data from included studies using a standardized extraction form. Any disagreements were resolved by discussion. The data extracted included the type of study, the number and characteristics of patients receiving ACEIs/ARBs, and mortality and severe AEs associated with COVID-19.

Severe AEs were defined as intensive care unit admission or the need for invasive or noninvasive ventilation. Studies reporting severe AEs based on information from the Chinese Center for Disease Control and Prevention^13^ were included. To avoid double-counting of patients in studies reporting multiple severe AE outcomes, we included the outcome with the largest number of patients in our analyses. For instances in which distinct data for ACEIs/ARBs were available, an aggregate was used given the small likelihood of combined receipt of both drugs.

The Newcastle-Ottawa Scale,^14^ a 9-point measure assessing the quality of cohort studies and case-control studies or case series, was used to evaluate the observational studies included. The Cochrane Risk of Bias 2 tool was used to assess the risk of bias in randomized clinical trials.^15^

### Statistical Analysis

For each outcome, a random-effects model was used to compare the odds ratios (ORs) and 95% CIs between patients who did and did not receive ACEIs/ARBs using Review Manager software, version 5.3 (Nordic Cochrane Center), and OpenMeta[Analyst] software, version 10.12 (Center for Evidence Synthesis, Brown University).^16^ For studies reporting hazard ratios (HRs), those HRs were converted to ORs using methodology defined in the *Cochrane Handbook for Systematic Reviews of Interventions*.^17^ Results from studies were grouped according to a prespecified variable (patients with hypertension [hypertension subgroup] vs patients with multiple mixed comorbidities [mixed subgroup]), and a series of subgroup analyses were performed. We also conducted a sensitivity analysis, in which studies reporting HRs (which were converted to ORs) were excluded to assess the robustness of results.

Statistical heterogeneity was assessed using the *I*^2^ statistic. Potential publication bias was assessed using funnel plots. The statistical significance threshold was *P* < .05.

## Results

Our search identified 1788 records from the PubMed and Embase databases; after removal of duplicates, 1664 records were screened, and 71 articles underwent full-text evaluation (eFigure 1 in the Supplement). Of those, 52 studies (40 cohort studies, 6 case series, 4 case-control studies, 1 randomized clinical trial, and 1 cross-sectional study) with 101 949 total participants met inclusion criteria and were included in the meta-analysis. The cohorts and methodological characteristics of the studies are described in the Table. Ten studies were ranked as having moderate quality and 41 studies were ranked as having high quality based on the Newcastle-Ottawa Scale (eTable in the Supplement). Funnel plots indicated no substantial publication biases (eFigure 2 and eFigure 3 in the Supplement).

**Table.** Baseline Characteristics of Included Studies

Source	Study type and location	Participants receiving ACEIs or ARBs, No.	Total participants, No.	Participant age, y	Male sex, %	Participants with hypertension, %	Participant clinical characteristics, %	Outcomes measured	Adjustment methods
Amat-Santos et al,^18^ 2020	Ongoing open-label RCT (RASTAVI), Spain	5	11	Median (IQR), 86 (84-88)	54.5	54.5	CKD: 36.4 Diabetes: 18.2 CAD: 18.2	Mortality	NA
Conversano et al,^19^ 2020	Retrospective case series, Italy	68	96	NR	NR	100	Hypertension: 100 CHD: 28.1 Diabetes: 22.9 Heart failure: 8.3	Mortality; median follow-up of 28 d	NA
Bae et al,^20^ 2020	Retrospective cohort study, US	78	590	NR	48.8	25.4	Diabetes: 26.3 CAD: 5.3 Heart failure: 3.6	Mortality and critical severity (ICU admission)	Multivariate logistic regression with propensity matching for age and comorbidities (hypertension, dyslipidemia, diabetes or prediabetes, CAD, heart failure, CVA, chronic lung disease, and CKD or ESRD)
Bean et al,^21^ 2020	Prospective multicenter cohort study, United Kingdom	399	1200	Mean (SD), 68 (17)	57.2	53.8	Diabetes: 34.8 IHD: 13.3 Heart failure: 8.9	Mortality and critical severity (ICU admission); follow-up of 21 d	Multivariate logistic regression adjusted for age, sex, and comorbidities (hypertension, diabetes, IHD, heart failure, and CKD)
Bravi et al,^7^ 2020	Retrospective case-control study, Italy	450	543	Mean (SD), 58 (21)	47.3	33.9	Diabetes: 12.1 COPD: 6.0	Very severe or lethal (ICU admission or death)	Multivariate logistic regression adjusted for age, sex, and comorbidities (diabetes, major cardiovascular diseases, COPD, cancer, and renal diseases)
Cannata et al,^22^ 2020	Prospective cohort study, Italy	173	397	NR	NR	NR	NR	Mortality; follow-up until death or discharge	Multivariate logistic regression adjusted for age, BMI, comorbidities (diabetes, COPD, LVEF<50%, and cancer), vital parameters, and laboratory values within 24 h after admission
Chen et al,^23^ 2020	Retrospective, cohort study, China	355	1182	Median (IQR), 68 (60-75)	49.1	100	Diabetes: 22.1 CHD: 17.4 CKD: 5.3	Mortality and critical severity (need for IMV); follow-up of 45 d	Multivariable Cox proportional hazards regression analyses adjusted for age, sex, comorbidities (CHD and diabetes), laboratory findings (creatinine levels), and receipt of various medications
Chen et al,^24^ 2020	Retrospective cohort study, China	81	312	Median (IQR), 69 (61-77)	55.1	93.9	CAD: 25.6	Mortality	Multivariable logistic regression models adjusted for clinically relevant parameters (age, sex, comorbidities, and laboratory findings) that differed between the 2 groups
Chen et al,^25^ 2020	Retrospective cohort study, China	32	71	NR	NR	NR	Diabetes: 100 Hypertension: 100	Mortality and clinical outcome of discharge or death in hospital	NA
Hippisley-Cox et al,^26^ 2020	Prospective cohort study, United Kingdom	4281	19 486	Mean (SD), 62 (21)	48.12	38.9	CVD: 18.2 COPD: 7.3 Diabetes: 7.0	Critical severity (ICU admission)	Cox proportional hazards models using imputed analysis including all exposure and explanatory variables
Felice et al,^9^ 2020	Prospective cohort study, Italy	82	133	NA	64.7	100	Diabetes: 25.6 Heart failure: 18.0 COPD: 10.5	Mortality and critical severity (ICU admission); mean follow-up of 15.8 d	Multivariable logistic regression adjusted for age, sex, BMI, days with duration of symptoms before admission, and comorbidities (previous cardiovascular events, diabetes, and cancer)
Feng et al,^4^ 2020	Retrospective cohort study, China	33	113	NR	NR	100	NR	Mortality and critical severity based on CCDC report	NA
Fosbol et al,^27^ 2020	Retrospective cohort study, Denmark	895	4480	NR	47.9	18.8	COPD:14.2 Diabetes: 9.2 CVD: 9.0 Heart failure: 5.4	Mortality and critical severity (ICU admission); median follow-up of 34 d	Cox regression adjusted for sex, highest obtained education, income, comorbidities (myocardial infarction, heart failure, CKD, stroke, peripheral artery disease, AF, diabetes, COPD, and cancer) and various medications
Gao et al,^28^ 2020	Retrospective cohort study, China	183	850	Mean (SD), 64 (11)	52.1	100	Diabetes: 26.8 COPD: 1.3 Heart failure: 1.2	Mortality and critical severity based on CCDC report	Cox proportional hazards model adjusted for age, sex, medical history (diabetes and IHD), sex, RAAS inhibitor (ACEI or ARB), insulin-treated diabetes, myocardial infarction, treatment by PCI or CABG, renal failure, chronic heart failure, asthma, COPD, and stroke
Gormez et al,^29^ 2020	Retrospective cohort study, Turkey	49	247	NR	62.3%	31.6%	Diabetes: 39.7% COPD: 5.7% CKD: 4.0% Heart failure: 0.8%	Critical severity (ICU admission); median follow-up of 13 d (ICU) and 7 d (other)	Bayesian logistic regression adjusted for age, sex, D-dimer level, neutrophil to lymphocyte ratio, CRP, and history of hypertension
Grasselli et al,^30^ 2020	Retrospective cohort study, Italy	NR	1608	NR	NR	100%	NR	Mortality	Multivariable Cox proportional hazards regression models including baseline characteristics, comorbidities, medications, and physiological variables at admission
Guo et al,^31^ 2020	Retrospective case series, China	19	187	Mean (SD), 59 (15)	48.7	32.6	Diabetes: 15.0 CHD: 11.2	Mortality	NA
Hu et al,^64^ 2020	Retrospective cohort study, China	65	149	Median (IQR), 57 (50-66)	59.1	100	Diabetes: 20.1 CKD: 4.0 COPD: 1.3	Mortality and critical severity (ICU admission)	NA
Huang et al,^32^ 2020	Prospective cohort study, China	20	50	NR	54.0	100	Diabetes: 8.0 COPD: 2.0	Mortality and critical severity based on CCDC report	NA
Hwang et al,^33^ 2020	Retrospective cohort study, South Korea	13	103	Mean (SD), 68 (15)	50.5	55.3	Diabetes: 34.0 CKD: 16.5 CVD: 11.7	Mortality	NA
Iaccarino et al,^34^ 2020	Cross-sectional study, Italy	655	1591	Mean (SD), 67 (0.4)	64.0	54.9	Diabetes: 16.9 CAD: 13.6 Heart failure: 11.8 CKD: 5.5	Mortality	NA
Jung et al,^35^ 2021	Prospective study (part of COVIP), Germany	157	324	Median (IQR), 75 (70-93)	69.1	65.1	Diabetes: 29.4 Heart failure: 14.1	Mortality	Multivariate logistic regression with propensity matching for age, BMI, sex, sequential organ failure assessment score, comorbidities (heart failure, IHD, renal insufficiency, chronic pulmonary disease, hypertension, and diabetes)
Jung et al,^36^ 2020	Retrospective cohort study, Korea	377	1954	NR	NR	NR	NR	Mortality and critical severity (need for IMV)	Multivariate logistic regression analysis adjusted for age, sex, Charlson Comorbidity Index score, immunosuppression, and hospital type
Khan et al,^37^ 2020	Prospective cohort study, United Kingdom	27	88	Mean (SD), 72 (14)	56.8	100	NR	Mortality and critical severity (ICU admission); follow-up for 60 d or until discharge or death	NA
Lam et al,^38^ 2020	Retrospective cohort study, US	335	614	NR	55.0	100	Diabetes: 40.7 CKD: 15.4 COPD: 13.4 Heart failure: 13.4	Mortality and critical severity (ICU admission)	NA
Li et al,^39^ 2020	Retrospective case series, China	115	362	Median (IQR), 66 (59-73)	53.2	100	Diabetes: 35.2% CVD: 18.8% CHD: 17.1% Heart failure: 2.8%	Mortality and critical severity based on CCDC report	NA
Liabeuf et al,^40^ 2020	Retrospective cohort study, France	96	268	Median (IQR), 73 (61-84)	58.2	56.7	Diabetes: 20.5 COPD: 9.7 CKD: 7.1	Mortality and critical severity (ICU admission)	Multivariate logistic regression model adjusted for age, sex, BMI, and CHD
Liu et al,^41^ 2021	Retrospective single-center case series, China	74	157	NR	46.5	100	Diabetes: 27.3 CAD: 10.2	Critical severity based on CCDC criteria	NA
Lopez-Otero et al,^42^ 2020	Retrospective single-center cohort study, Spain	210	965	Mean (SD), 60 (20)	43.9	30.9	Diabetes: 12.8 CAD: 4.4	Mortality and critical severity (ICU admission)	Multivariate logistic regression models adjusted for variables with *P* < .05 in the univariate analysis (fever, oxygen saturation <95%, age, sex, obesity, health personnel, and dependency status)
Mancia et al,^43^ 2020	Case–control study, Italy	2896	6272	Mean (SD), 68 (13)	63.3	NR	CHD: 7.5 Heart failure: 5.1	Critical severity (assisted ventilation) and fatal infection	NA
Matsuzawa et al,^44^ 2020	Retrospective cohort study, Japan	21	39	Mean (SD), 71 (12)	69.2	100	Diabetes: 35.9 CKD: 7.7 COPD: 2.6 Heart failure: 2.6	Mortality and critical severity (ICU admission)	Multivariate logistic regression model adjusted for age, sex, and presence of diabetes
Mehta et al,^6^ 2020	Retrospective cohort study, US	212	1735	NR	NR	NR	NR	Mortality and critical severity (ICU admission)	Propensity score estimated using multivariable logistic regression model adjusted for age, sex, and comorbidities (hypertension, diabetes, heart failure, COPD, and CAD)
Meng et al,^45^ 2020	Retrospective cohort study, China	17	42	Median (IQR), 65 (56-69)	57.1	100	CHD: 19.0 Diabetes: 14.2	Mortality	NA
Mostaza et al,^46^ 2020	Retrospective cohort study, Spain	192	404	Mean (SD), 85 (5)	54.7	73.8	Diabetes: 28.0 Heart failure: 18.8 CKD: 15.6	Mortality; follow-up until death or discharge	NA
Oussalah et al,^47^ 2020	Retrospective longitudinal cohort study, France	44	149	Median (IQR), 65 (54-77)	61.0	49.6	Diabetes: 28.6 COPD: 11.3 CKD: 6.0	Mortality and critical severity (ICU admission and IMV)	NA
Pan et al,^48^ 2020	Retrospective cohort study, China	41	282	Median (IQR), 69 (62-76)	50.7	100	Diabetes: 27.2 CVD: 7.8 COPD: 2.8	Mortality and critical severity (ICU admission)	NA
Reynolds et al,^49^ 2020	Retrospective cohort study, US	1293	2141	NR	NR	100	NR	Severity (ICU admission, need for assisted ventilation, or death)	Multivariable logistic regression adjusted for demographic characteristics and comorbidities
Richardson et al,^50^ 2020	Case series, US	456	1366	NR	NR	100	NR	Mortality and critical severity (ICU admission); median follow-up of 4 d	NA
Rossi et al,^51^ 2020	Prospective cohort study, Italy	818	2653	NR	50.1	18.1	Diabetes: 12.0 Heart failure: 5.8 COPD: 5.4 CKD: 2.5	Mortality	Multivariate proportional hazards models adjusted for age, sex, and Charlson Comorbidity Index score
Sardu et al,^52^ 2020	Prospective cohort study, Italy	45	62	Mean (SD), 58 (18)	66.1	100	Diabetes: 25.8 COPD: 16.1 CVD: 11.3	Mortality and critical severity (ICU admission)	NA
Selcuk et al,^5^ 2020	Retrospective cohort study, Turkey	74	113	NR	52.2	100	Diabetes: 42.5 CAD: 24.8 Heart failure: 8.0	Mortality and critical severity (ICU admission)	Multivariate logistic regression analysis adjusted for age, CAD, receipt of ACEIs or ARBs, and laboratory findings (WBC count and D-dimer, creatinine, plasma glucose, and lactate dehydrogenase levels)
Senkal et al,^53^ 2020	Retrospective cohort study, Turkey	165	248	NR	NR	100	NR	Mortality and critical severity (ICU admission)	Cases matched to controls according to age, sex, number of days ill before hospital admission, comorbidities (diabetes, COPD or asthma, CAD, chronic heart failure, and CKD), current smoking status, and various medications
Shah et al,^54^ 2020	Retrospective cohort study, US	207	531	NR	41.1	80.0	Diabetes: 42.9 Chronic heart failure: 14.9 COPD: 7.5 CAD: 7.0	Mortality and critical severity (ICU admission)	Multivariable logistic regression analysis adjusted for age, sex, BMI, baseline comorbidities, and presenting illness severity
Tan et al,^55^ 2020	Retrospective cohort study, China	31	100	NR	51.0	100	Diabetes: 28.0 CHD: 18.0	Mortality and critical severity (ICU admission)	NA
Tedeschi et al,^56^ 2020	Prospective cohort study, Italy	175	311	Median (IQR), 76 (67-83)	72.3	100	CVD: 42.1 Diabetes: 23.8 COPD: 15.8	Mortality	Multivariate Cox regression analysis adjusted for age, sex, presence of comorbidities, and COPD
Trifiro et al,^57^ 2020	Retrospective cohort study, Italy	9522	42 926	Median (IQR), 69 (57-79)	62.6	13.1	Diabetes: 18.0 COPD: 3.5 Heart failure: 6.5	Mortality and critical severity (ICU admission)	Mixed-effects Cox proportional hazards model adjusted for center, age, sex, Charlson Comorbidity Index score, drug receipt, and comorbidities (pneumonia and influenza, IHD, AF, heart failure, hypertension, CVD, diabetes, liver disease, dementia, renal failure, COPD, cancer, and rheumatic diseases)
Xu et al,^58^ 2020	Retrospective cohort study, China	40	101	Median (IQR), 65 (58-73)	52.5	100	Diabetes: 18.8 COPD: 2.0 Heart failure: 1.0	Mortality and critical severity (ICU admission)	Multivariable analysis with logistic model adjusted for age and sex
Yang et al,^59^ 2020	Retrospective case-control study, China	43	126	Median (IQR), 66 (61-73)	49.2	100	Diabetes: 30.2	Death and severity based on CCDC report	NA
Yuan et al,^60^ 2020	Retrospective cohort study, China	196	733	NR	NR	100	NR	Mortality	Propensity score estimated using multivariable logistic regression adjusted for age, sex, history of hypertension, chronic heart disease, diabetes, tumor, COPD, chronic liver disease, CKD, and baseline vital signs
Zhang et al,^61^ 2020	Retrospective cohort study, China	188	1128	Median (IQR), 64 (56-69)	53.5	100	Diabetes: 21.3 CHD: 11.6 CVD: 3.6 COPD: 0.5	Mortality and critical severity (ICU admission); follow-up of 28 d	Cox proportional hazards model adjusted for age, sex, comorbidities (CHD, CKD, CVD, and diabetes), and in-hospital medications
Zhou et al,^10^ 2020	Retrospective cohort study, China	989	3572	NR	NR	NR	NR	Mortality; follow-up of 28 d	Propensity score matching for age, sex, disease severity, comorbidities, and receipt of calcium channel blocker medication
Zhou et al,^8^ 2020	Retrospective case series, China	15	36	Mean (SD), 65 (10)	52.8	100	Diabetes: 25.0 CVD: 19.4	Mortality and transfer to high-level hospital	Multivariate logistic regression adjusted for age, sex, hospitalization time, and time from onset to hospital admission

Abbreviations: ACEI, angiotensin-converting enzyme inhibitor; AF, atrial fibrillation; ARB, angiotensin receptor blocker; BMI, body mass index (calculated as weight in kilograms divided by height in meters squared); CABG, coronary artery bypass graft; CAD, coronary artery disease; CCDC, Chinese Center for Disease Control and Prevention; CHD, coronary heart disease; CKD, chronic kidney disease; COPD, chronic obstructive pulmonary disease; COVIP, Corona Virus Disease in Very Elderly Intensive Care Patients study; CRP, C-reactive protein; CVA, cardiovascular accident; CVD, cerebrovascular disease; ESRD, end-stage renal disease; ICU, intensive care unit; IHD, ischemic heart disease; IMV, invasive mechanical ventilation; IQR, interquartile range; LVEF, left ventricular ejection fraction; NA, not applicable; NR, not reported; PCI, percutaneous coronary intervention; RAAS, renin-angiotensin-aldosterone system; RASTAVI, Remodeling After Transcatheter Aortic Valve Implantation clinical trial; RCT, randomized clinical trial; WBC, white blood cell.

Most studies included in the meta-analysis were retrospective^20,21,33,44^ or observational^4,6,26,37,61,62,63^ and were conducted in China,^4,8,10,23,24,25,28,31,32,35,39,41,45,48,55,58,59,60,62,64,65,66^ Europe,^5,7,9,18,19,22,30,34,40,43,46,47,51,52,53,56,57^ or North America.^6,20,49,50,54^ Two studies^22,38^ included a subgroup of patients receiving ACEIs/ARBs that were explicitly discontinued during hospital admission. The results from this subgroup of patients were included in the group of patients receiving ACEIs/ARBs and compared with those not receiving ACEIs/ARBs. In studies in which both multivariate and propensity-matched scores were reported,^28,42,61^ data from the multivariate analyses were used. A total of 26 545 of 101 949 patients (26.0%) overall and 4813 of 11 696 patients (41.2%) in the hypertension subgroup were receiving ACEIs/ARBs (Table).

### Mortality

**Figure 1.**
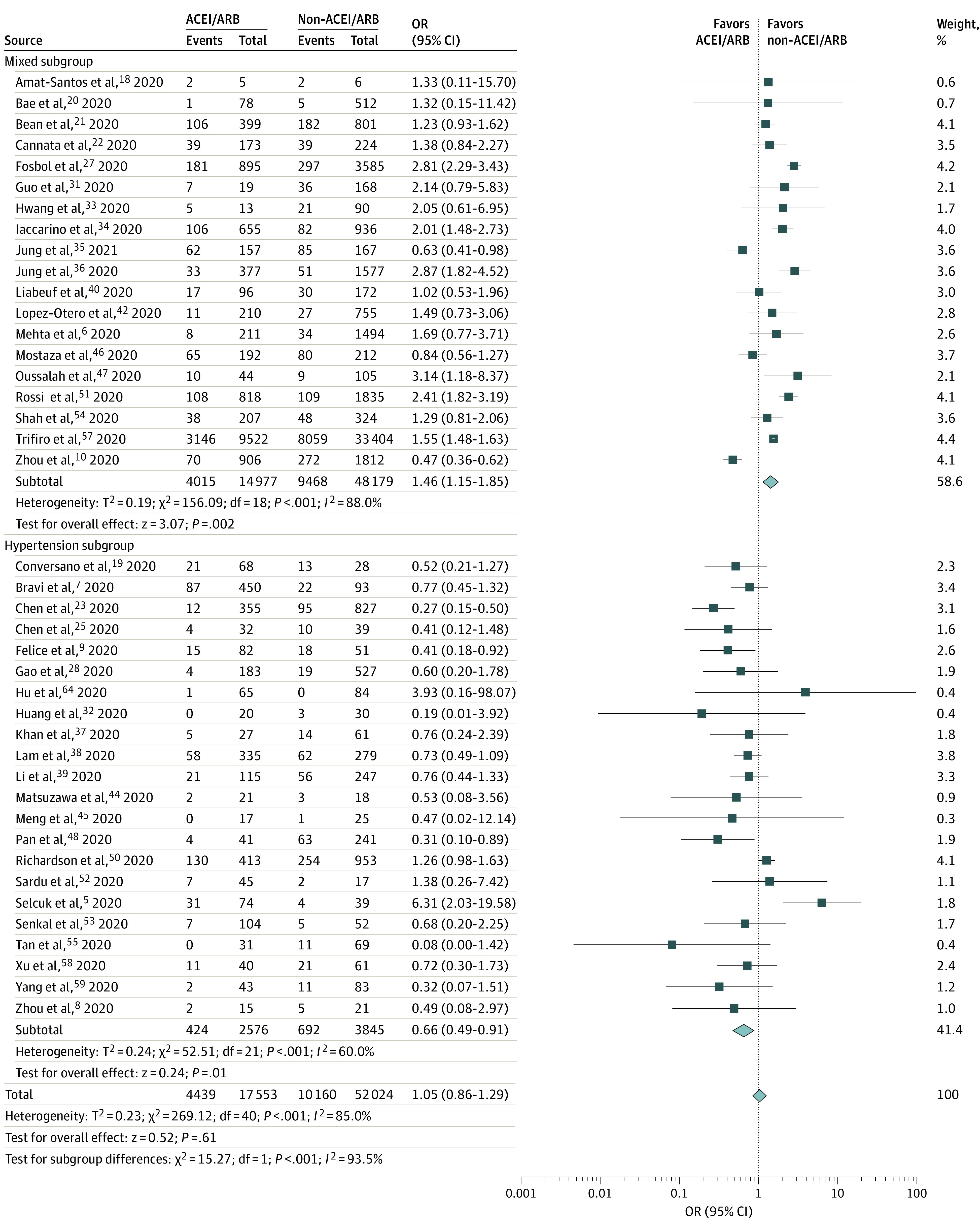
Subgroup Analysis of Unadjusted Mortality Among Patients Who Did and Did Not Receive ACEIs or ARBs Subgroup analysis of mortality in 41 studies of patients who did and did not receive ACEIs or ARBs. A total of 19 studies included a mixed subgroup (a sample population with multiple mixed comorbidities), and 22 studies included a hypertension subgroup (a sample population with hypertension). Diamonds represent 95% CIs for subtotal and total ORs. ACEI indicates angiotensin-converting enzyme inhibitor; ARB, angiotensin receptor blocker; and OR, odds ratio.

A total of 41 studies (69 577 total participants) that compared mortality rates of patients receiving vs not receiving ACEIs/ARBs were included in the meta-analysis. Overall, the results of the pooled unadjusted meta-analysis indicated no increases in the risk of death among those who received ACEIs/ARBs (unadjusted OR, 1.05; 95% CI, 0.86-1.29; *P* = .61; *I*^2^ = 85.0%) compared with those who did not (Figure 1). The subgroup analysis revealed significant reductions in mortality among patients in the hypertension subgroup who were receiving ACEIs/ARBs (unadjusted OR, 0.66; 95% CI, 0.49-0.91; *P* = .01). In contrast, the mixed subgroup comprising patients with multiple comorbidities indicated significant increases in mortality among those receiving ACEIs/ARBs (unadjusted OR, 1.46; 95% CI, 1.15-1.85; *P* = .002)

**Figure 2.**
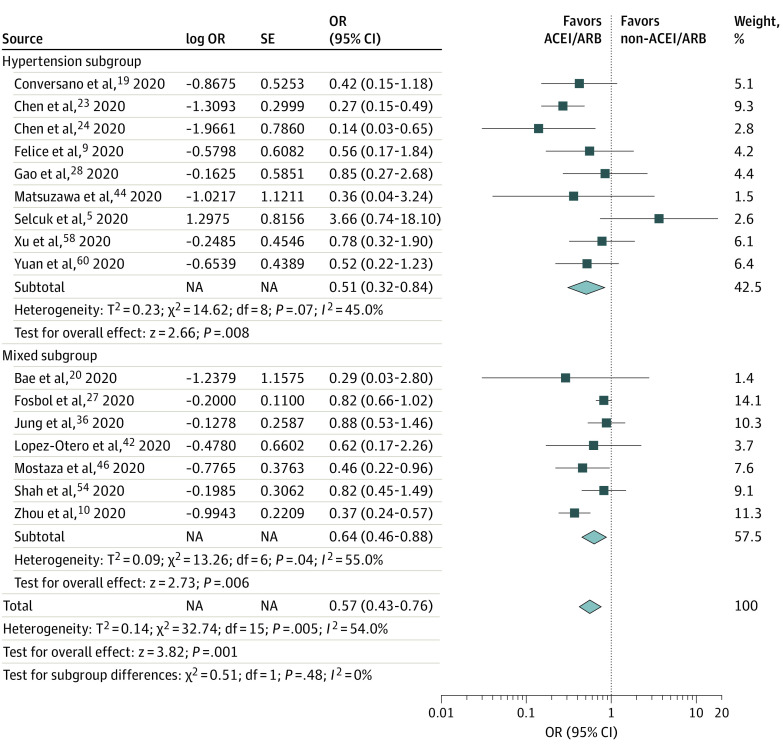
Subgroup Analysis of Adjusted Mortality Among Patients Who Did and Did Not Receive ACEIs or ARBs Subgroup analysis of adjusted mortality in 16 studies of patients who did and did not receive ACEIs or ARBs. A total of 7 studies included a mixed subgroup (a sample population with multiple mixed comorbidities), and 9 studies included a hypertension subgroup (a sample population with hypertension). Diamonds represent 95% CIs for subtotal and total ORs. ACEI indicates angiotensin-converting enzyme inhibitor; ARB, angiotensin receptor blocker; and OR, odds ratio.

However, a pooled analysis of 17 studies (17 392 total participants) using an adjusted analysis of mortality found reductions in the risk of death among patients receiving vs not receiving ACEIs/ARBs (adjusted OR [aOR], 0.57; 95% CI, 0.43-0.76; *P* < .001; *I*^2^ = 54.0%) (Figure 2). A significant decrease in the risk of death was observed in both subgroups (for the hypertension subgroup, aOR, 0.51 [95% CI, 0.32-0.84]; *P* = .008; for the mixed subgroup, aOR, 0.64 [95% CI, 0.46-0.88]; *P* = .006).

###  Severe Adverse Events

**Figure 3.**
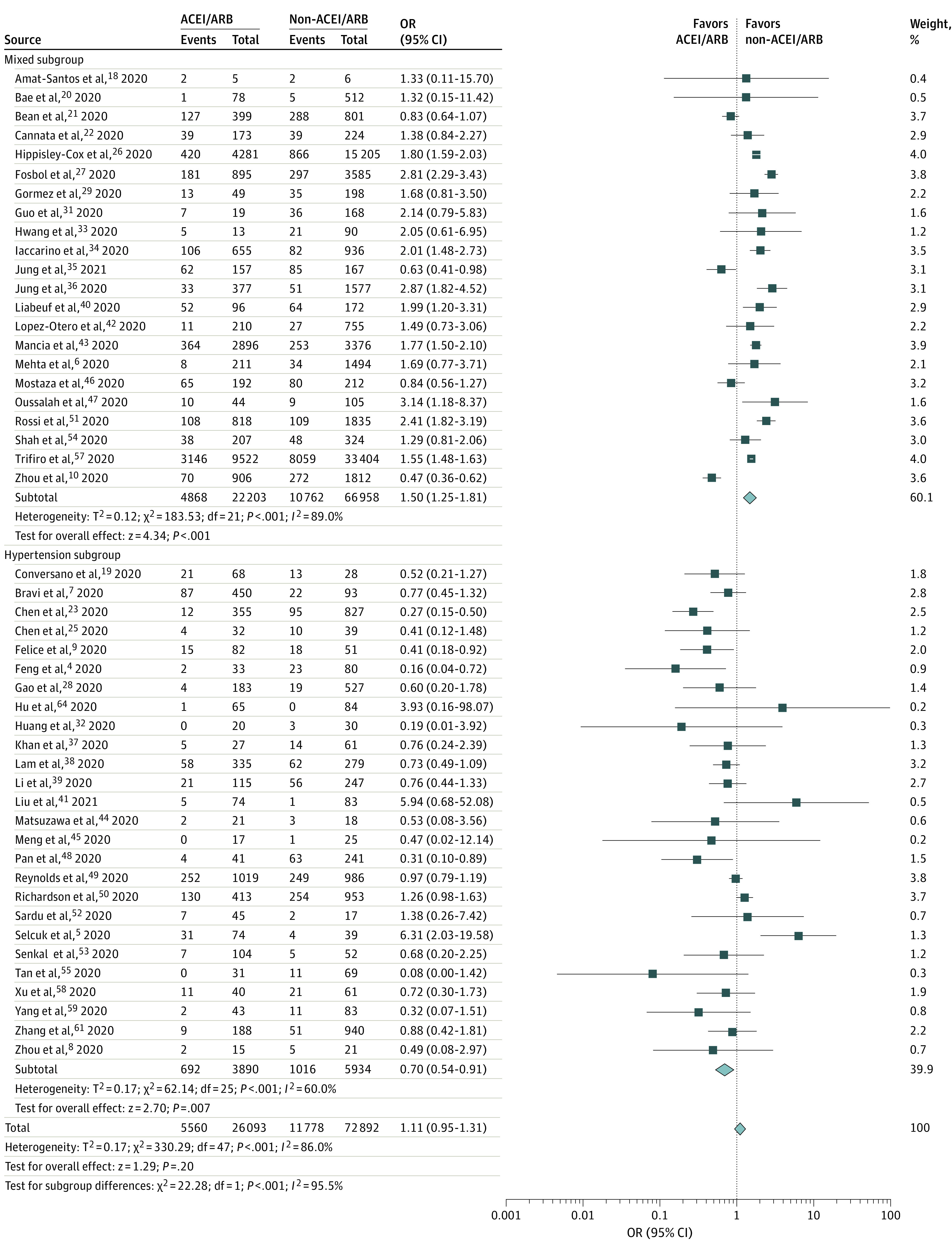
Subgroup Analysis of Unadjusted Mortality and Severe Adverse Events Among Patients Who Did and Did Not Receive ACEIs or ARBs Subgroup analysis of mortality and severe adverse events in 48 studies of patients who did and did not receive ACEIs or ARBs. A total of 22 studies included a mixed subgroup (a sample population with multiple mixed comorbidities), and 26 studies included a hypertension subgroup (a sample population with hypertension). Diamonds represent 95% CIs for subtotal and total ORs. ACEI indicates angiotensin-converting enzyme inhibitor; ARB, angiotensin receptor blocker; and OR, odds ratio.

Unadjusted values for severe AEs were reported in 48 studies that included a total of 98 985 participants. A pooled analysis found comparable results among patients who did and did not receive ACEIs/ARBs (unadjusted OR, 1.11; 95% CI, 0.95-1.31; *P* = .20; *I*^2^ = 86.0%) (Figure 3). Notably, the 26 studies including a hypertension subgroup (unadjusted OR, 0.70; 95% CI, 0.54-0.91; *P* = .007) and the 33 studies including a mixed subgroup (unadjusted OR, 1.50; 95% CI, 1.25-1.81; *P* < .001) reported statistically significant results.

**Figure 4.**
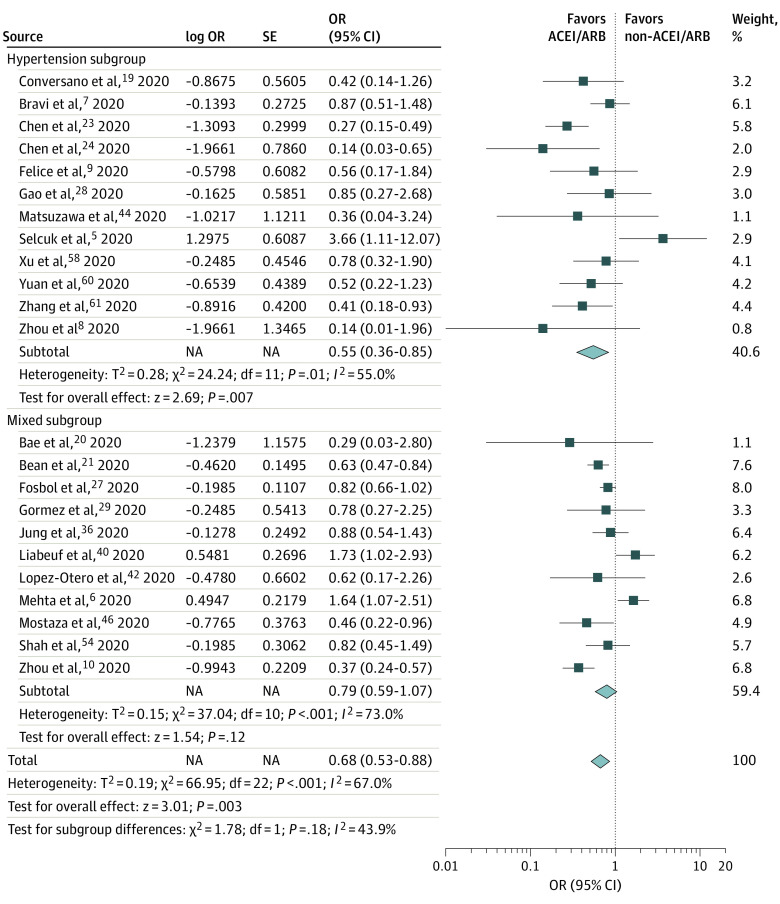
Subgroup Analysis of Adjusted Mortality and Severe Adverse Events Among Patients Who Did and Did Not Receive ACEIs or ARBs Subgroup analysis of adjusted mortality and severe adverse events in 23 studies of patients who did and did not receive ACEIs or ARBs. A total of 11 studies included a mixed subgroup (sample population with multiple mixed comorbidities), and 12 studies included a hypertension subgroup (defined as a sample population with hypertension). Diamonds represent 95% CIs for subtotal and total ORs. ACEI indicates angiotensin-converting enzyme inhibitor; ARB, angiotensin receptor blocker; and OR, odds ratio.

A total of 23 studies (23 129 total participants) reported an adjusted risk of severe AEs associated with the receipt of ACEIs/ARBs in a COVID-19 cohort. The adjusted covariates for each study are listed in the Table. A significant decrease in severe AEs was found in patients who received ACEIs/ARBs compared with those who did not (aOR, 0.68; 95% CI, 0.53-0.88; *P* = .003; *I*^2^ = 67.0%) (Figure 4). This reduced risk remained significant among the hypertension subgroup in 12 studies (aOR, 0.55; 95% CI, 0.36-0.85; *P* = .007). However, in the mixed subgroup, the decreased risk was not statistically significant (OR, 0.79; 95% CI, 0.59-1.07; *P* = .12). A sensitivity analysis that excluded studies reporting HRs indicated statistically significant results for mortality but nonsignificant results for severe AEs (eFigure 4 and eFigure 5 in the Supplement). Subgroup analyses of studies of moderate quality (OR, 0.36; 95% CI, 0.25-0.51; *P* < .001) and high quality (OR, 0.78; 95% CI, 0.60-1.00; *P* = .05) indicated reduced risk of adjusted severe AEs among both hypertension and mixed subgroups (eFigure 6 in the Supplement).

## Discussion

The results of this systematic review and meta-analysis of 52 studies with 101 949 total patients indicated a significant association between the receipt of ACEIs/ARBs and reductions in mortality and severe AEs among patients in the hypertension subgroup. In the mixed subgroup of patients with multiple comorbidities, this association was observed only when the analysis was adjusted for significant covariates.

Our results are consistent with those of another meta-analysis^67^ comprising 18 studies and 17 311 patients with hypertension. This previous meta-analysis reported a lower risk (risk ratio, 0.84; 95% CI, 0.73-0.95; *P* = .007) of the composite outcome (death, intensive care unit admission, mechanical ventilation, and progression to severe or critical pneumonia) among patients receiving ACEIs/ARBs. The present meta-analysis extends this finding to patients with multiple mixed comorbidities, suggesting that ACEIs/ARBs may have a substantial protective role in COVID-19 outcomes across all patient groups.

Notably, the protective implications of ACEIs/ARBs in the mixed subgroup were observed only after adjustments to potential and important confounders, such as age and comorbidities. This finding suggests that comorbidities may have an important role in COVID-19 clinical outcomes and that ACEIs/ARBs might be associated with further improvements in potential outcomes. In a large retrospective cohort study by Fosbol et al^27^ that included 4480 patients with COVID-19, an unadjusted analysis indicated worse outcomes among those who received ACEIs/ARBs. However, after multivariate adjustments, this finding was no longer statistically significant. Similar results were observed in multiple small retrospective cohort studies.^29,36,42,54^ It is worth noting that most studies included in the meta-analyses were retrospective and observational; with these study designs, unmeasured confounding factors and potential biases are inevitable. In addition, patients receiving ACEIs/ARBs are more likely to have heart failure, cardiovascular disease, hypertension, and comorbidities, which are associated with an increased risk of death among patients with COVID-19.^3^ Therefore, it is necessary to adjust for these confounders when evaluating the protective benefits of ACEIs/ARBs for mortality and severe AEs.

The potential mechanisms underlying the beneficial consequences of ACEIs/ARBs remain unknown. Our results suggest that these benefits are not solely associated with better blood pressure control, as patients receiving antihypertensive medications that were not ACEIs/ARBs had comparably inferior clinical outcomes in the adjusted subgroup analysis. Concerns about upregulation of angiotensin-converting enzyme 2 receptors with the receipt of RAAS inhibitors, which are derived from inconsistent results in studies with small samples,^68,69^ have been challenged by reports of deactivation of RAAS^61^ with chronic receipt of ACEIs/ARBs. Such downregulation may limit the inflammatory process, reducing acute lung injury among patients with COVID-19.^49^

Nevertheless, the findings of the present meta-analysis are consistent with those of national and international scientific experts,^70,71,72^ who recommend continuation of ACEIs/ARBs unless they are clinically contraindicated. This meta-analysis also indicated that, after adjustment for case mix, patients with hypertension and COVID-19 who received ACEIs/ARBs were 0.55 times as likely to experience a severe AE than those who did not receive ACEIs/ARBs, with a similar extent of benefit observed in the combined hypertension and mixed comorbidities subgroups. Although our study clarifies the association between RAAS inhibitors and mortality among patients with COVID-19, future randomized clinical trials are warranted to establish causality.

### Limitations

This study has limitations. First, the study was limited by the insufficient data and varying study designs available, which did not allow for comparison of these analyses with a control group. The meta-analysis was primarily composed of observational studies because studies with higher levels of evidence, such as randomized clinical trials, were lacking. Second, the meta-analysis indicated substantial unadjusted and moderate adjusted levels of heterogeneity, which is typical in observational studies that include patients with diverse characteristics across large geographic regions. Nevertheless, measures were taken to maintain a homogeneous study population. A standard definition for severe AEs was used, and patients with unconfirmed COVID-19 were excluded. Third, we did not define the criteria for chronic receipt of ACEIs/ARBs. Insufficient description was available to distinguish between study participants, which is likely a factor associated with the increased heterogeneity observed in the study. With these limitations in mind, there were no data indicating that the receipt of ACEIs/ARBs was associated with harm if patients subsequently contracted COVID-19; on the contrary, ACEIs/ARBs may be associated with substantial protective benefits.

## Conclusions

This comprehensive systematic review and meta-analysis of 52 studies indicated no higher risks of multivariable-adjusted mortality or severe AEs associated with the receipt of ACEIs/ARBs, which is consistent with recommendations for the continuation of these medications among patients for whom they are prescribed for the treatment of any condition. On the contrary, ACEIs and ARBs may be associated with protective benefits, particularly among patients with hypertension. Future randomized clinical trials are warranted to confirm the beneficial implications of these medications.

## Notes

Supplement. **eMethods.** Search Strategy in PubMed **eTable.** Quality Assessment of Included Clinical Trials: Newcastle Ottawa Scale for Nonrandomized Clinical Trials and Cochrane Collaboration Tool for Assessing Risk of Bias in Randomized Clinical Trials **eFigure 1.** Preferred Reporting Items for Systematic Reviews and Meta-Analyses (PRISMA) Flow Diagram of the Clinical Trial Selection Process **eFigure 2.** Funnel Plot for Unadjusted Critical or Fatal Outcomes for Patients With COVID-19 Receiving ACEIs or ARBs **eFigure 3.** Funnel Plot for Unadjusted Death for Patients With COVID-19 Receiving ACEIs or ARBs **eFigure 4.** Sensitivity Analysis Excluding Studies Reporting Hazard Ratios for Critical or Fatal Outcomes **eFigure 5.** Sensitivity Analysis Excluding Studies Reporting Hazard Ratios for Mortality Outcomes **eFigure 6.** Subgroup Analysis of Studies With Moderate or High Quality for Adjusted Critical or Fatal Outcomes
