# Supplementary material for: Association Between Renin-Angiotensin-Aldosterone System Inhibitors and Clinical Outcomes in Patients With COVID-19: A Systematic Review and Meta-analysis
Source: JAMA Netw Open. 2021 Mar 31;4(3):e213594. doi: 10.1001/jamanetworkopen.2021.3594 (PMC8013817; doi:10.1001/jamanetworkopen.2021.3594)
Supplement: Supplement. — eMethods. Search Strategy in PubMed eTable. Quality Assessment of Included Clinical Trials: Newcastle Ottawa Scale for Nonrandomized Clinical Trials and Cochrane Collaboration Tool for Assessing Risk of Bias in Randomized Clinical Trials eFigure 1. Preferred Reporting Items for Systematic Reviews and Meta-Analyses (PRISMA) Flow Diagram of the Clinical Trial Selection Process eFigure 2. Funnel Plot for Unadjusted Critical or Fatal Outcomes for Patients With COVID-19 Receiving ACEIs or ARBs eFigure 3. Funnel Plot for Unadjusted Death for Patients With COVID-19 Receiving ACEIs or ARBs eFigure 4. Sensitivity Analysis Excluding Studies Reporting Hazard Ratios for Critical or Fatal Outcomes eFigure 5. Sensitivity Analysis Excluding Studies Reporting Hazard Ratios for Mortality Outcomes eFigure 6. Subgroup Analysis of Studies With Moderate or High Quality for Adjusted Critical or Fatal Outcomes [file jamanetwopen-e213594-s001.pdf]

Supplementary Online Content

Baral R, Tsampasian V, Debski M, et al. Association between renin-angiotensin-aldosterone system inhibitors and clinical outcomes in patients with COVID-19: a systematic review and meta-analysis. *JAMA Netw Open*. 2021;4(3):e213594.
doi:10.1001/jamanetworkopen.2021.3594

eMethods. Search Strategy in PubMed

eTable. Quality Assessment of Included Clinical Trials: Newcastle Ottawa Scale for Nonrandomized Clinical Trials and Cochrane Collaboration Tool for Assessing Risk of Bias in Randomized Clinical Trials

eFigure 1. Preferred Reporting Items for Systematic Reviews and Meta-Analyses (PRISMA) Flow Diagram of the Clinical Trial Selection Process

eFigure 2. Funnel Plot for Unadjusted Critical or Fatal Outcomes for Patients With COVID-19 Receiving ACEIs or ARBs

eFigure 3. Funnel Plot for Unadjusted Death for Patients With COVID-19 Receiving ACEIs or ARBs

eFigure 4. Sensitivity Analysis Excluding Studies Reporting Hazard Ratios for Critical or Fatal Outcomes

eFigure 5. Sensitivity Analysis Excluding Studies Reporting Hazard Ratios for Mortality Outcomes

eFigure 6. Subgroup Analysis of Studies With Moderate or High Quality for Adjusted Critical or Fatal Outcomes

This supplementary material has been provided by the authors to give readers additional information about their work.

© 2021 Baral R et al. *JAMA Netw Open*. All rights reserved.

eMethods. Search Strategy in PubMed

1. Angiotensin converting enzyme [All Fields]
2. Angiotensin receptor blocker* [All Fields]
3. Renin angiotensin aldosterone system [All Fields]
4. ACEi [All Fields]
5. ARB [All Fields]
6. RAAS [All Fields]
7. perindopril [mesh] OR Lisinopril [mesh] OR enalapril [mesh] OR captopril [mesh]
8. olmesartan [mesh] OR candesartan [mesh] OR losartan [mesh] OR telmisartan [mesh]
9. 1 OR 2 OR 3 OR 4 OR 5 OR 6 OR 7 OR 8
10. Coronavirus [All Fields]
11. covid-19 [All Fields]
12. 2019-n-COV [All Fields]
13. SARS-CoV-2 [All Fields]
14. 2019-nCOV [All Fields]
15. 10 OR 11 OR 12 OR 13 OR 14
16. 9 AND 15

© 2021 Baral R et al. *JAMA Netw Open*. All rights reserved.

eTable. Quality Assessment of Included Clinical Trials: Newcastle Ottawa Scale for Nonrandomized Clinical Trials and Cochrane Collaboration Tool for Assessing Risk of Bias in Randomized Clinical Trials

Non-RCT					
Authors	Selection	Comparability	Outcome/Exposures	Total	Quality of study
Andrea	***	*	**	6	Moderate
Bae	***	**	**	7	High
Bean	****	**	**	8	High
Bravi	****	**	***	9	High
Cannata	***	**	**	7	High
Chen C	***	**	**	7	High
Chen F	**	**	**	6	Moderate
Chen Y	****	**	**	8	High
Cox	****	**	***	9	High
Felice	***	**	**	7	High
Feng	****	**	**	8	High
Fosbol	***	**	**	7	High
Gao	***	**	**	7	High
Gormez	***	**	**	7	High
Grasseli	***	**	**	7	High
Guo	***	*	**	6	Moderate
Hu	***	**	**	7	High
Huang	***	**	**	7	High
Hwang	***	**	**	7	High
Iaccarino	****	**	**	8	High

© 2021 Baral R et al. *JAMA Netw Open*. All rights reserved.

Jung C	***	**	**	7	High
Jung S	***	**	**	7	High
Khan	***	**	**	7	High
Lam	***	*	**	6	Moderate
Li	***	*	**	6	Moderate
Liabeuf	***	**	**	7	High
Liu	****	**	*	7	High
Lopez-Otero	***	*	**	6	Moderate
Mancia	****	**	**	8	High
Matsuzawa	***	**	**	7	High
Mehta	****	*	**	7	High
Meng	***	**	**	7	High
Mostaza	***	**	**	7	High
Oussalah	***	**	**	7	High
Pan	***	**	**	7	High
Reynolds	****	**	**	8	High
Richardson	***	*	**	6	Moderate
Rossi	****	**	***	9	High
Sardu	***	**	**	7	High
Selcuk	***	**	**	7	High
Senkal	***	**	**	7	High
Shah	***	**	**	7	High
Tan	***	*	**	6	Moderate
Tedeschi	***	**	**	7	High

© 2021 Baral R et al. *JAMA Netw Open*. All rights reserved.

Trifiro	***	**	**	7	High
Xu	***	**	**	7	High
Yang	****	**	**	8	High
Yuan	***	**	**	7	High
Zhang	***	**	**	7	High
Zhou F	**	**	**	6	Moderate
Zhou X	***	**	*	6	Moderate
RCT					
Amat-Santos#	Domain 1<br>Domain 2<br>Domain 3<br>Domain 4<br>Domain 5<br>Overall				Some concerns<br>Some concerns<br>Low<br>Low<br>Low<br>Some concerns

*Each star indicates a point for each component. Any study can obtain a maximum of four, two and three stars for each component (selection, comparability, outcomes) respectively. Trials with a total score of 7 or higher are considered to be high-quality studies, and those lower than 5 are considered as low-quality studies. These scores indicate at least moderate quality of the included trials.
#Cochrane risk of bias 2 tool was used to assess the risk of bias in the randomised trial.

© 2021 Baral R et al. *JAMA Netw Open*. All rights reserved.

eFigure 1. Preferred Reporting Items for Systematic Reviews and Meta-Analyses (PRISMA) Flow Diagram of the Clinical Trial Selection Process

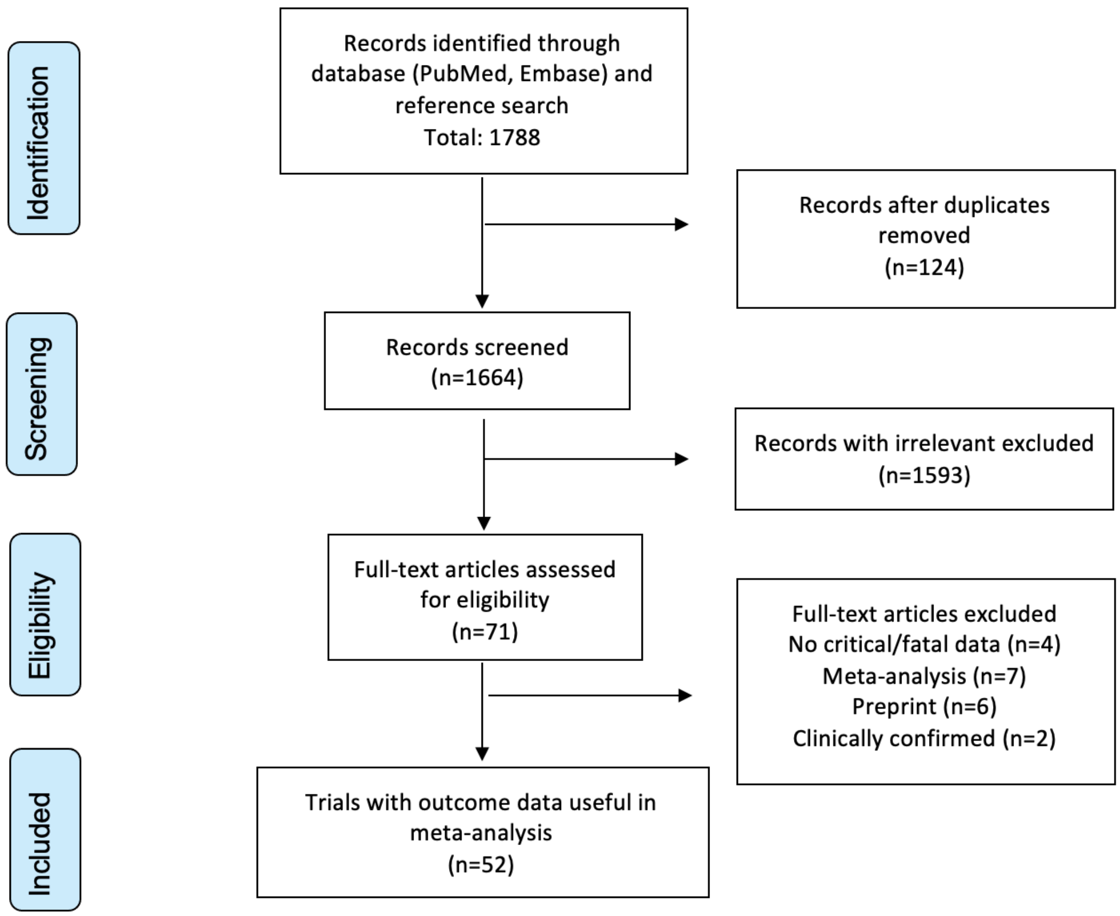

© 2021 Baral R et al. *JAMA Netw Open*. All rights reserved.

eFigure 2. Funnel Plot for Unadjusted Critical or Fatal Outcomes for Patients With COVID-19 Receiving ACEIs or ARBs

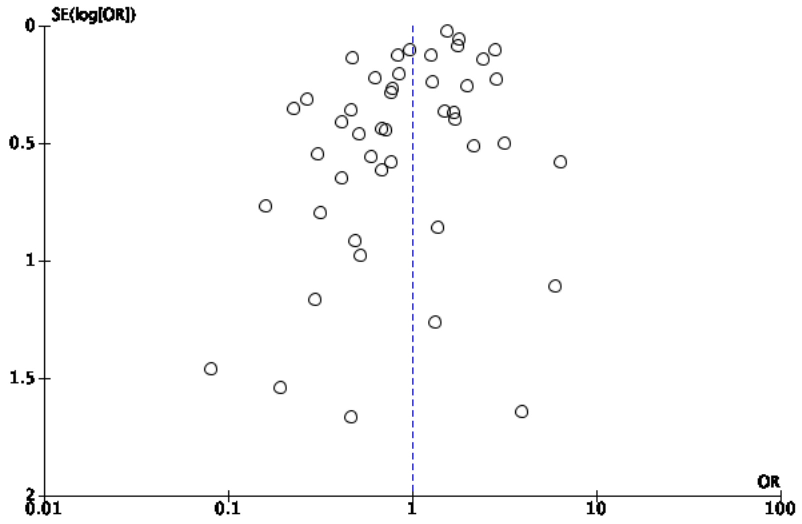

Funnel plot for unadjusted critical or fatal outcomes for patients with COVID-19 receiving ACEIs or ARBs indicating no substantial publication bias.

© 2021 Baral R et al. *JAMA Netw Open*. All rights reserved.

eFigure 3. Funnel Plot for Unadjusted Death for Patients With COVID-19 Receiving ACEIs or ARBs

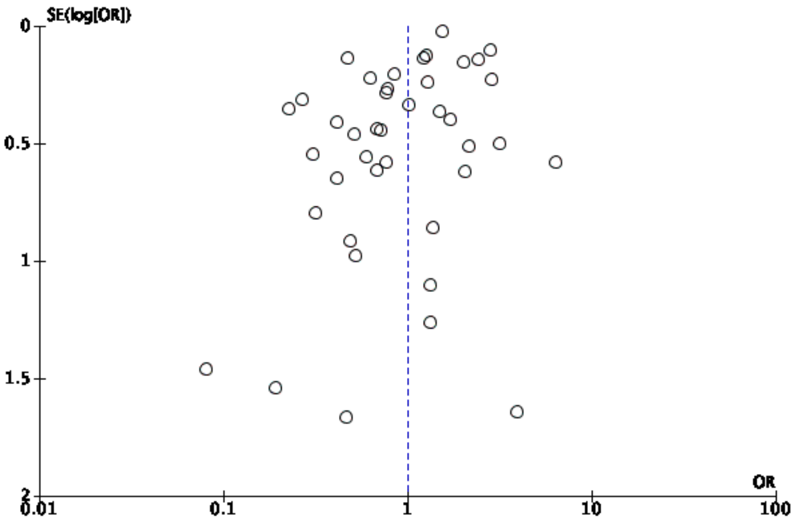

Funnel plot for unadjusted mortality outcome for patients with COVID-19 receiving ACEIs or ARBs indicating no substantial publication bias.

© 2021 Baral R et al. *JAMA Netw Open*. All rights reserved.

eFigure 4. Sensitivity Analysis Excluding Studies Reporting Hazard Ratios for Critical or Fatal Outcomes

**Studies**	Estimate (95% C.I.)
Bae 2020	0.294 (0.030, 2.897)
Bean 2020	0.630 (0.472, 0.840)
Bravi 2020	0.870 (0.508, 1.490)
Cannata 2020	0.050 (0.005, 0.540)
Chen F 2020	0.136 (0.035, 0.532)
Felice 2020	0.560 (0.171, 1.830)
Gormez 2020	0.780 (0.272, 2.240)
Jung S 2020	0.880 (0.538, 1.440)
Liabeuf 2020	1.730 (1.020, 2.934)
Lopez-Otero 2020	0.620 (0.170, 2.260)
Matsuzawa 2020	0.360 (0.037, 3.530)
Mehta 2020	1.640 (1.072, 2.510)
Mostaza 2020	0.459 (0.222, 0.949)
Selcuk 2020	3.660 (0.737, 18.180)
Shah 2020	0.820 (0.448, 1.500)
Xu 2020	0.780 (0.315, 1.930)
Yuan 2020	0.523 (0.224, 1.220)
Zhou X 2020	0.140 (0.009, 2.210)
**Overall (I^2=6093 % , P< 0.001)**	**0.744 (0.548, 1.011)**

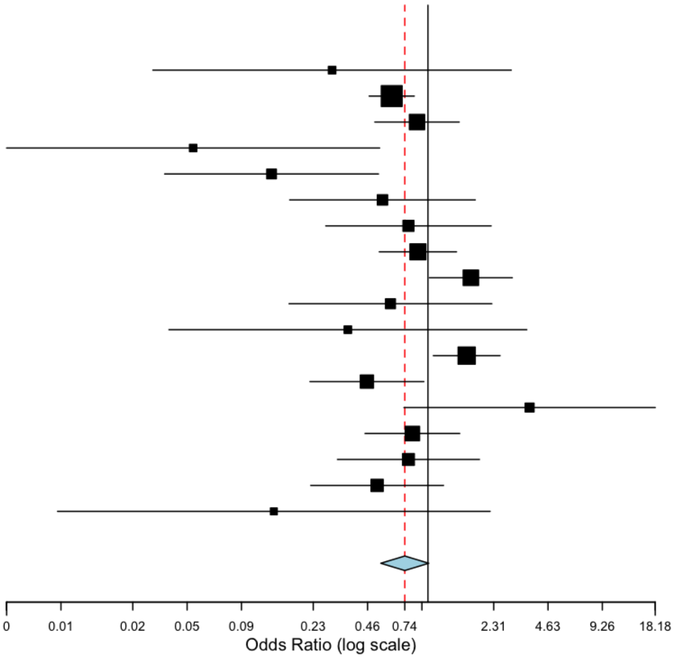

Sensitivity analysis excluding studies reporting hazard ratios for critical or fatal outcomes (OR, 0.744; 95% CI, 0.548-1.011; *P* = .059).

© 2021 Baral R et al. *JAMA Netw Open*. All rights reserved.

eFigure 5. Sensitivity Analysis Excluding Studies Reporting Hazard Ratios for Mortality Outcomes

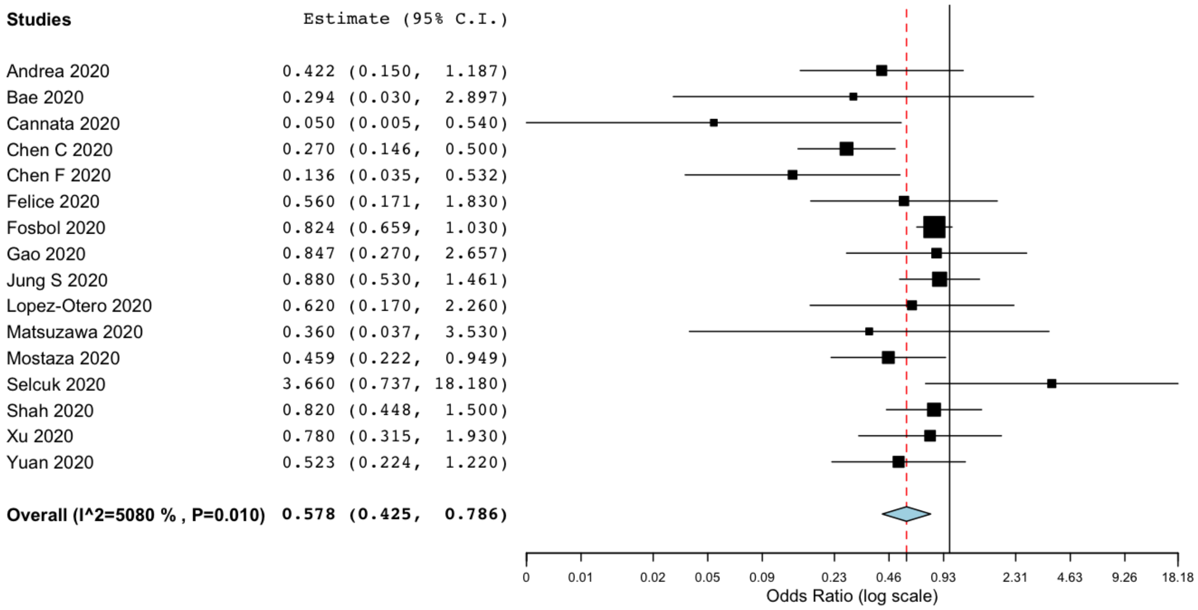

Sensitivity analysis excluding studies reporting hazard ratios for mortality outcomes (OR, 0.578; 95% CI, 0.425-0.786; *P* < .001).

© 2021 Baral R et al. *JAMA Netw Open*. All rights reserved.

eFigure 6. Subgroup Analysis of Studies With Moderate or High Quality for Adjusted Critical or Fatal Outcomes

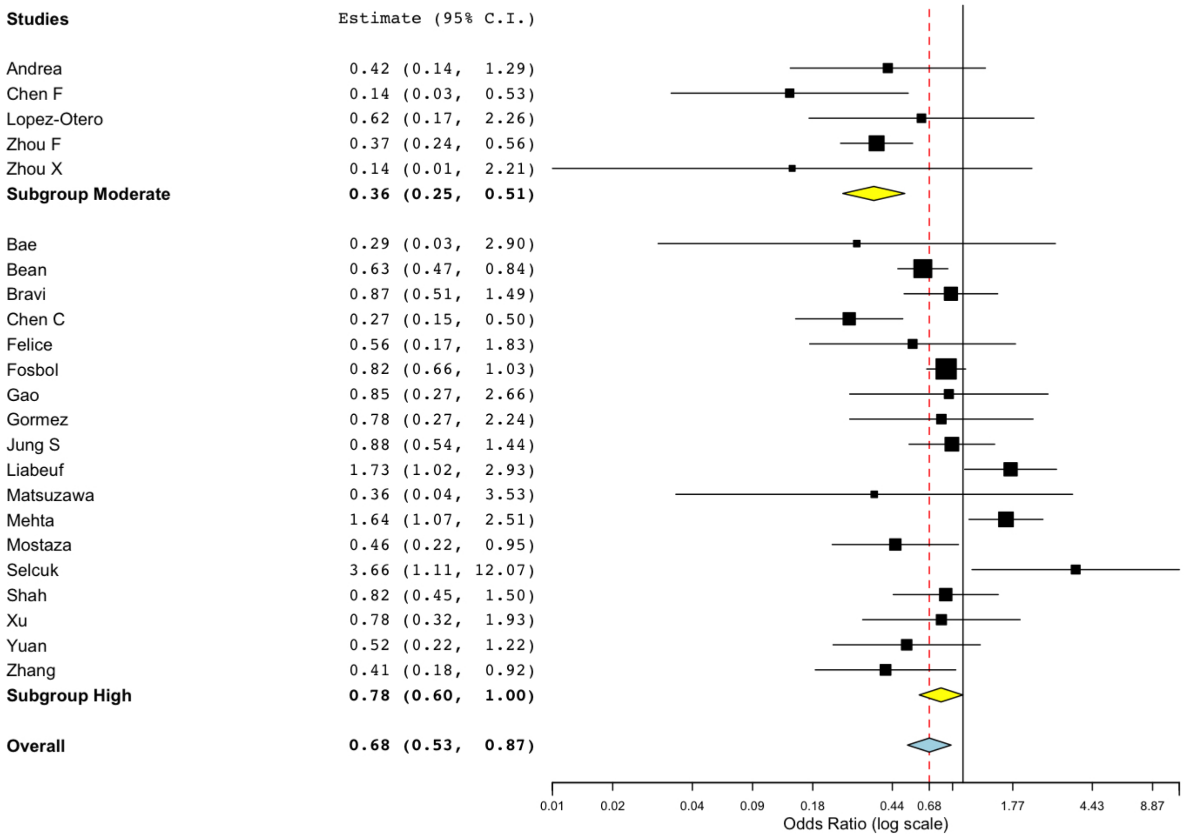

Subgroup analysis of studies with moderate (OR, 0.36; 95% CI, 0.25-0.51; *P* < .01) and high quality (OR, 0.78; 95% CI, 0.60-1.00; *P* = .05) indicated reduced association for adjusted critical or fatal outcomes in both groups.

© 2021 Baral R et al. *JAMA Netw Open*. All rights reserved.
